# Brief Bursts Self-Inhibit and Correlate the Pyramidal Network

**DOI:** 10.1371/journal.pbio.1000473

**Published:** 2010-09-07

**Authors:** Thomas K. Berger, Gilad Silberberg, Rodrigo Perin, Henry Markram

**Affiliations:** 1 Laboratory of Neural Microcircuitry, Brain Mind Institute, Ecole Polytechnique Fédérale de Lausanne (EPFL), Lausanne, Switzerland; 2 Department of Neuroscience, Karolinska Institute, Stockholm, Sweden; European Brain Research Institute, Italy

## Abstract

Inhibitory pathways are an essential component in the function of the neocortical microcircuitry. Despite the relatively small fraction of inhibitory neurons in the neocortex, these neurons are strongly activated due to their high connectivity rate and the intricate manner in which they interconnect with pyramidal cells (PCs). One prominent pathway is the frequency-dependent disynaptic inhibition (FDDI) formed between layer 5 PCs and mediated by Martinotti cells (MCs). Here, we show that simultaneous short bursts in four PCs are sufficient to exert FDDI in all neighboring PCs within the dimensions of a cortical column. This powerful inhibition is mediated by few interneurons, leading to strongly correlated membrane fluctuations and synchronous spiking between PCs simultaneously receiving FDDI. Somatic integration of such inhibition is independent and electrically isolated from monosynaptic excitation formed between the same PCs. FDDI is strongly shaped by I(h) in PC dendrites, which determines the effective integration time window for inhibitory and excitatory inputs. We propose a key disynaptic mechanism by which brief bursts generated by a few PCs can synchronize the activity in the pyramidal network.

## Abstract 2

A multi-cell patch clamp study reveals the summation properties of frequency-dependent disynaptic inhibition between neocortical pyramidal cells and shows how brief bursts of activity in a few cells can synchronize the entire microcircuit.

## Author Summary

The neocortex of the mammalian brain contains many more excitatory neurons than inhibitory neurons, yet inhibitory neurons are essential components of neocortical circuitry. Inhibitory neurons form dense and intricate connections with excitatory neurons, which are mainly pyramidal cells. One prominent pathway formed between pyramidal cells and inhibitory Martinotti cells is frequency-dependent disynaptic inhibition (FDDI), which mediates a strong inhibitory signal in the microcircuitry of the neocortex. Here, we reveal deeper insight into how FDDI is mediated and recruited within the circuit, showing that short simultaneous bursts in four pyramidal cells are sufficient to exert FDDI in all neighboring pyramidal cells within the dimensions of a cortical column. As few as three synchronous action potentials in three pyramidal cells can trigger FDDI. This powerful inhibition is mediated by only a few inhibitory neurons yet correlates membrane potential fluctuations, leading to synchronous spiking between pyramidal cells that simultaneously receive FDDI. The inhibitory signals are independent and electrically isolated from excitation mediated by neighboring PCs via basal dendrites. We propose FDDI as an important pathway that is readily activated by brief bursts of action potentials and correlates neocortical network activity.

## Introduction

The mammalian neocortex consists of neurons that form an intricate network of recurrent circuits [1]–[3]. The synaptic wiring between cells follows a number of stereotypic rules including targeting specific domains of neurons, specific connection probabilities, target neuron preferences, and specific short-term synaptic dynamics [1]–[5]. Revealing these rules is essential to understand the mechanisms that generate the response of a cortical column (or functional unit) to any external input. In particular, it is crucial to identify the synaptic pathways that enable the neocortex to appropriately respond to all possible environmental stimuli.

Neocortical neurons receive excitatory and inhibitory inputs over a variety of different network activity states [6] that seem to be proportionally regulated [7]. This balanced excitatory and inhibitory activity is remarkable since the large majority of cells in the neocortex are (excitatory) pyramidal cells (PCs), only around 25% are inhibitory GABAergic interneurons [8],[9], and almost 90% of the neocortical synapses are presumably excitatory [10]. This relatively small population of interneurons is responsible for generating a precisely matched inhibition for a variety of cortical network states. One synaptic principle for dynamically adjusting the level of excitation within a neocortical column is the use of dynamically depressing excitatory synapses [11]–[13], but how inhibitory synaptic pathways ensure dynamic application of balanced inhibition as a function of the moment-to-moment excitation of the neocortical column is not clear.

A disynaptic pathway and dynamic circuit mechanism allowing an activity-dependent recruitment of inhibition was recently reported: frequency-dependent disynaptic inhibition (FDDI) between PCs is indeed a common pathway in multiple cortical areas that is dynamically regulated by the firing rate and the number of presynaptic PCs [14]–[17]. In contrast to many other cortical connections, the PC–Martinotti cell (MC) synapse is strongly facilitating. In response to high frequency stimulation of a PC, spiking activity of MCs can be recruited, thus providing a level of inhibition that depends on the previous excitation level in the network. MCs display a characteristic ascending axonal arborization up to layer 1 [18], and they are the only interneurons that target the combination of oblique, apical, and tuft dendrites of their neighboring PCs [3],[14]. FDDI has so far been explored mainly as a pairwise interaction between PCs and MCs, but little is known about how this synaptic pathway could operate to dynamically apply inhibition to the microcircuit as a function of multi-cellular activity.

Here, we used multi-neuron whole cell recordings to characterize *summation properties* of FDDI between layer 5 thick tufted PCs within the dimensions of a neocortical column. FDDI tends to summate linearly with coincident excitatory postsynaptic potentials (EPSPs) from neighboring PCs but may also shunt some input arriving at the apical dendrite. Three to four PCs firing simultaneously are sufficient to generate FDDI in all PCs within the dimensions of a cortical column, and eight to nine PCs can saturate the amount of hyperpolarization recorded from their somata. A brief, high frequency burst in only a few PCs can therefore constitute a gating mechanism for further excitatory input to the apical dendrites of the entire column. This inhibition promotes subthreshold correlations and synchronous spiking in PCs.

## Results

In order to study the network properties of FDDI, we obtained simultaneous whole-cell recordings from neighboring thick tufted layer 5 PCs and in some cases also layer 5 MCs. In total, 1,185 PCs and 14 MCs in 283 clusters from 133 animals were recorded for this study.

### I_h_ Located in Postsynaptic PCs Spatiotemporally Separates Synaptic Inputs

**Figure 1:**
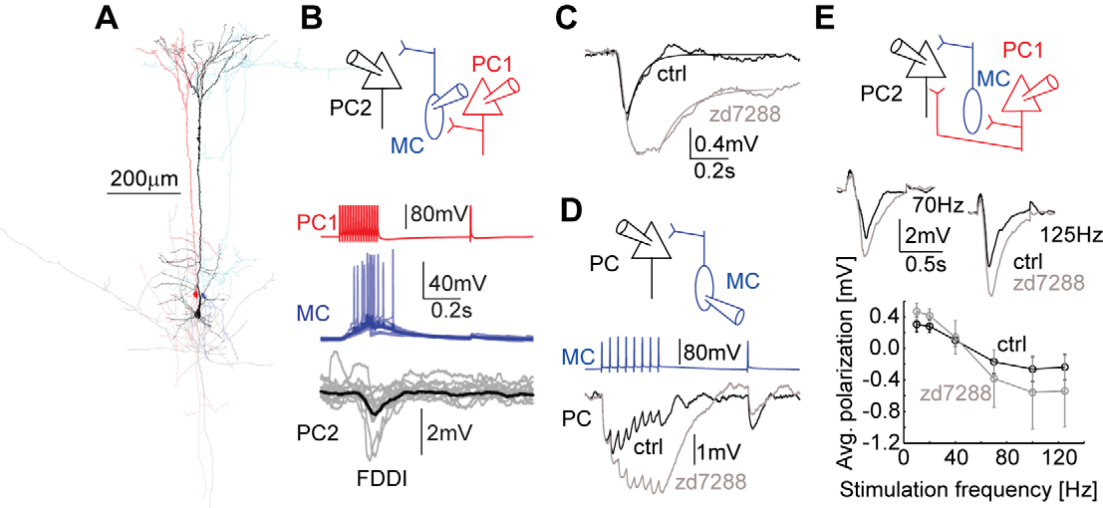
Components of frequency-dependent disynaptic inhibition (FDDI) and its modulation by I_h_ currents in PCs. (A) Reconstruction of the complete pathway of FDDI (2 PCs in black and red, 1 MC in blue). (B) Top, sketch of the FDDI pathway. Bottom, stimulation (70 Hz, 15 APs) of the presynaptic PC (in red) leads to delayed AP firing in the MC (in blue), giving in turn a hyperpolarizing inhibitory signal in the postsynaptic PC (in black). Single repetitions are in faint, mean traces in full colors. (C) Stimulation of a presynaptic PC eliciting FDDI in a postsynaptic PC before (black) and after (gray) bath application of 50 µM I_h_ blocker zd7288. (D) Stimulation of a presynaptic MC (8 APs at 20 Hz plus a single AP 0.5 s later) and the corresponding responses in a postsynaptic PC before (black) and after (gray) bath application of 50 µM zd7288. (E) Top, stimulation of a single (or multiple, not sketched) PC(s) (red) that target(s) a postsynaptic PC (black) with an excitatory direct connection as well as FDDI (via one or multiple unpatched MC(s)), before (black) and after (gray) bath application of 50 µM zd7288 (middle). The overall polarization of the postsynaptic PC (measured by the integral from EPSP onset to 0.5 s after train stimulation offset) depends on the stimulation frequency (stimulation train always contained 15 APs, only 70 and 125 Hz traces are shown in the middle, mean responses of 10–20 iterations). I_h_ block leads to a larger dynamic range of EPSP‐FDDI balance with respect to the stimulation frequency (bottom). Error bars (E) denote s.e.m.

Figure 1 illustrates the basic components (A,B) that mediate FDDI. A presynaptic PC (red) projecting onto an MC (blue) excites the MC using a strongly facilitating synapse, which in turn gives rise to a delayed inhibition in another postsynaptic PC (FDDI, black). Monosynaptic excitatory connections between PCs occurred in 14% of all tested cases (probability of occurrence was 0.14; 463 out of 3,342 tested connections), while PC-MC connections occurred far more frequently (0.43; 26/61) and MC-PC connections had a probability of occurrence of 0.31 (18/58). The entire FDDI loop occurs with a probability of 0.283 (859/3,041), which is more than double the monosynaptic connectivity between two PCs.

Silberberg and Markram (2007) previously showed a strong modulation of FDDI by I_h_ currents [14]. Blocking I_h_ currents with extracellular application of zd7288 leads to larger amplitudes (average 75% increase, *n* = 23, μ_ctrl_ = 0.99±0.5 mV, μ_zd7288_ = 1.73±0.99 mV, *p* = 0.0002, paired *t* test) and longer decay time constants (250% increase, μ_ctrl_ = 0.051±0.01 s, μ_zd7288_ = 0.182±0.071 s, *p* = 7.64e-9) of FDDI (Figure 1C). In some cases (3 out of 26) FDDI disappeared after I_h_ block. Since zd7288 blocks I_h_ irreversibly [19], we do not know whether the disappearance is due to a drug action or a general rundown. On the other hand, I_h_ block never leads to FDDI appearance de novo (*n* = 19). In order to understand whether the effects can be attributed to I_h_ on the intermediate interneuron or on the postsynaptic PC, we recorded from the entire disynaptic pathway while I_h_ was blocked. Facilitating EPSPs from PCs to MCs were only slightly changed in the presence of zd7288 (average 8% decrease of maximal depolarization; *n* = 5, μ_ctrl_ = 2.275±1.961 mV, μ_zd7288_ = 2.431±1.825 mV, *p* = 0.384, paired *t* test), whereas MC input onto PCs displayed increased synaptic summation (Figure 1D). Thus, the strong effect of zd7288 on FDDI is likely to be mediated by I_h_ in PCs.

PCs receiving both disynaptic inhibition and monosynaptic excitation from their neighboring PCs displayed the tendency of a frequency-dependent transition from a net depolarization to hyperpolarization (Figure 1E, *n* = 4). Blockage of I_h_ resulted in increased frequency dependence, enhancing both low-frequency depolarization and high-frequency hyperpolarization. Together with the observed shortening of synaptic events, this suggests that I_h_ in PCs acts to localize synaptic inputs, both spatially and temporally.

### Selective Non-Linear Summation of Excitatory Inputs with FDDI

**Figure 2:**
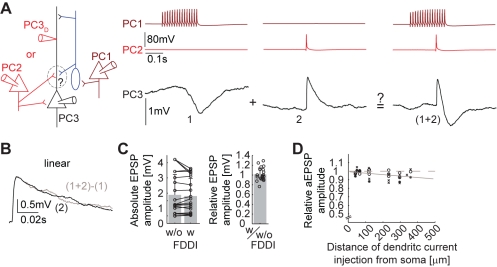
Selective non-linear summation of excitatory inputs with FDDI. (**A**) Sketch of the experimental setup and stimulation protocol. Left, PC 1 (dark red) connects via one or multiple unpatched MC(s) (blue) to PC 3 (black), giving rise to FDDI upon high frequency stimulation (left column, 15 APs at 70 Hz). PC 2 (red) is directly connected to PC 3 and triggers an EPSP upon stimulation (middle column, single AP, timed to be between the onset and the half-amplitude of FDDI). Alternatively, a patch electrode was positioned at the apical dendrite at various distances from the soma (PC3_D_, traces not shown). Right, synchronous stimulation (high frequency and single AP in PC 1 and 2, respectively) with the resulting response in PC 3. (B) Example of linear summation of EPSP and FDDI. The black trace shows a mean EPSP triggered by an AP in PC 2, and the gray trace is the subtraction of the EPSP-FDDI trace (synchronous stimulation of PC 1 and 2) minus the FDDI trace (stimulation of PC 1 only). (C) Comparison of the absolute EPSP amplitudes, in control condition (w/o FDDI) and with synchronously activated FDDI (w FDDI), and relative EPSP amplitude (ratio of EPSP amplitude with FDDI activation divided by control EPSP amplitude). (D) Distance dependence (distance between dendritic patch electrode and the soma) of the relative aEPSP amplitude, calculated as the ratio of the aEPSP amplitude with FDDI activation divided by control aEPSP amplitude without FDDI activation. For this experiment, an aEPSP was injected into PC 3_D_, and no PC 2 was stimulated. Linear fit was A = −0.0001695±0.000135 x+0.99329±0.028 (A, relative amplitude; x, distance from soma; 95% confidence interval). Note the broken ordinate. Error bars (C) denote s.d.

Monosynaptic excitation between PCs mainly targets their basal dendrites [20] while FDDI mainly targets their apical and tuft dendrites [14]. It is not clear to what extent these two inputs interact. We therefore activated both pathways simultaneously and quantified the linearity of summation. Clusters of three PCs, with a PC receiving FDDI from a neighboring PC and a direct excitatory connection from another PC, were stimulated in a way that FDDI and a direct EPSP coincided (Figure 2A).

We observed supra-, sub-, and linear amplitude summation in the soma (Figure 2B) in different experiments, and on average there was no significant difference in EPSP amplitude between control and coinciding FDDI (Figure 2C, *n* = 21, μ_ctrl_ = 1.885±1.334 mV, μ_FDDI_ = 1.808±1.154 mV, *p* = 0.295, paired *t* test). Inhibition in the distal dendrites may not shunt the peak amplitude of fast AMPA-mediated EPSPs from the basal dendrites but could reduce the total charge. We did not, however, observe any significant change in the integral of the EPSPs (μ_ctrl_ = 0.08±0.057 mV*ms, μ_FDDI_ = 0.074±0.048 mV*ms, *p* = 0.15, paired *t* test).

Next, we used the same protocol to investigate the summation of FDDI with excitatory input to the apical dendrite (Figure 2A). Instead of stimulating a neighboring PC, we synchronously injected a brief current (aEPSC) into the trunk of the apical dendrite (50–350 µm away from the soma) that mimicked EPSP kinetics (τ_rise_ = 0.5 ms, τ_decay_ = 2 ms) and peak amplitude (200–500 pA, tuned to match a somatic voltage depolarization of 1–4 mV). The somatic amplitude (Figure 2D) and integral of dendritic aEPSPs was slightly reduced by FDDI input in a distance dependent manner and as a function of the number of presynaptic PCs. We used fast AMPA kinetics for the aEPSPs, which might underestimate the shunting effect by FDDI on events with slower kinetics, namely NMDA components and EPSPs filtered by dendritic attenuation. A further technical limitation of artificial EPSPs via dendritic recording besides the focalization is the fact that excitatory synapses rather target spines, not the trunk like the patch electrode.

Nevertheless, these data suggest that FDDI is more effective in shunting synaptic input from the apical and tuft dendrites than input from the basal dendrites, revealing a dual and separable action between layer 5 PCs: direct excitation mostly onto basal dendrites, and indirect inhibition mostly onto the apical and tuft dendrites. This finding is supported by the anatomical separation of the inputs (Figure 1A, see also [3],[14],[20]).

### FDDI Mediated by Few MCs

**Figure 3:**
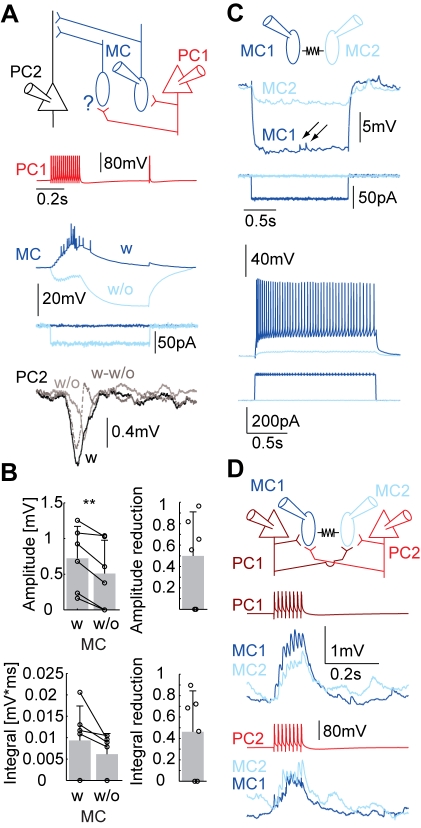
Few MCs mediate FDDI. (A) Sketch of the experimental setup and stimulation protocol. Top, PC 1 connects to an MC, which in turn inhibits PC 2. Possible other MCs (labeled with a question mark) might mediate FDDI between PC 1 and PC 2. Bottom, high frequency stimulation (15 APs at 70 Hz) of PC 1 (red) results in spiking in the postsynaptic MC in control condition (blue trace, average of 12 iterations), but only if the MC is not inhibited by a hyperpolarizing current step (light blue trace, −40 pA). The resulting FDDI in PC 2 is depicted in the bottom panel, with the control condition (black), the recorded MC inhibited (light gray), and the subtraction control – inhibited (dashed gray). In this example there was/were (an)other MC(s) mediating FDDI. (B) Absolute (left bar graph) and relative (right bar graph) comparison of FDDI amplitude (top panels) and integral (bottom panels) with and without the contribution of an FDDI-mediating MC. (C) Two MCs connected with a electrical synapses. A hyperpolarizing step current was injected into one cell (blue), leading to a (weaker) hyperpolarization in the electrically coupled cell (light blue). The arrows mark EPSPs in the blue cell that did not pass through the electrical synapses. A depolarizing step current injected into one cell leads to spiking (blue trace), but to a very mild depolarization in the coupled cell only (light blue trace). APs did not pass efficiently through electrical synapses. (D) The same two MCs were both postsynaptic to two PCs (top, sketch). Stimulation of PC1 (middle, dark red) or PC 2 (bottom, red) led to facilitating EPSPs in both of the MCs. Error bars (B) denote s.d.

We performed a set of experiments to estimate the number of MCs that meditate FDDI between two PCs. We stimulated a presynaptic PC that synapses onto an MC, which in turn projects to another PC (Figure 3A). Every other iteration, the MC was prevented from discharge by a hyperpolarizing step current, thereby isolating the effect of this one MC on the FDDI recorded in the postsynaptic PC. FDDI amplitude was reduced to 47.5%±38.1% (integral to 45.3%±35%) when the single MC was prevented from participating (Figure 3B, *n* = 7, amplitude: μ_wMC_ = 0.692±0.417 mV, μ_w/oMC_ = 0.460±0.446 mV, *p* = 0.0011, integral: μ_wMC_ = 0.08±0.082 mV*ms, μ_w/oMC_ = 0.053±0.049 mV*ms, *p* = 0.1148, paired *t* tests). These results show that although on average multiple MCs participate in FDDI, a single MC can make a significant contribution to the overall FDDI produced in a target PC. The exact number of intermediate MCs is not straightforward to extrapolate. Assuming linear amplitude summation of the MCs' inhibitory postsynaptic potentials (IPSPs), three MCs (μ_wMC_/(μ_wMC_−μ_w/oMC_)) participate on average in FDDI upon stimulation of one layer 5 PC (range 1–28 MCs). We might have indirectly prevented further neighboring MCs from spiking through electrical coupling by hyperpolarizing the recorded MC, which might have resulted in an underestimate of participating MCs. Figure 3C shows an example of two MCs coupled via electrical synapses. Their coupling coefficient was 0.11 for hyperpolarizing step currents, which is within the range that has been found in previous studies [21],[22]. Due to low pass filtering, miniature EPSPs in one MC do not pass to the other MC (arrows in Figure 3C). For the same reason, the coupling coefficient was only 0.02 for action potentials. Thus, electrical synapses can only play a role in the communication in the FDDI network if synaptic inputs summate with a sufficiently slow time constant so that the signal is not eliminated by low-pass filtering. The same two MCs were targeted by two PCs that were recorded at the same time (Figure 3D) providing direct evidence for PC-MC divergent and PC-MC convergent connectivity. We also found multiple cases of MC-PC divergent connectivity (data not shown), indicating that neighboring PCs might share a common pool of MCs for feed-forward and feed-back inhibition.

### FDDI Correlates Activity between Neighboring PCs

**Figure 4:**
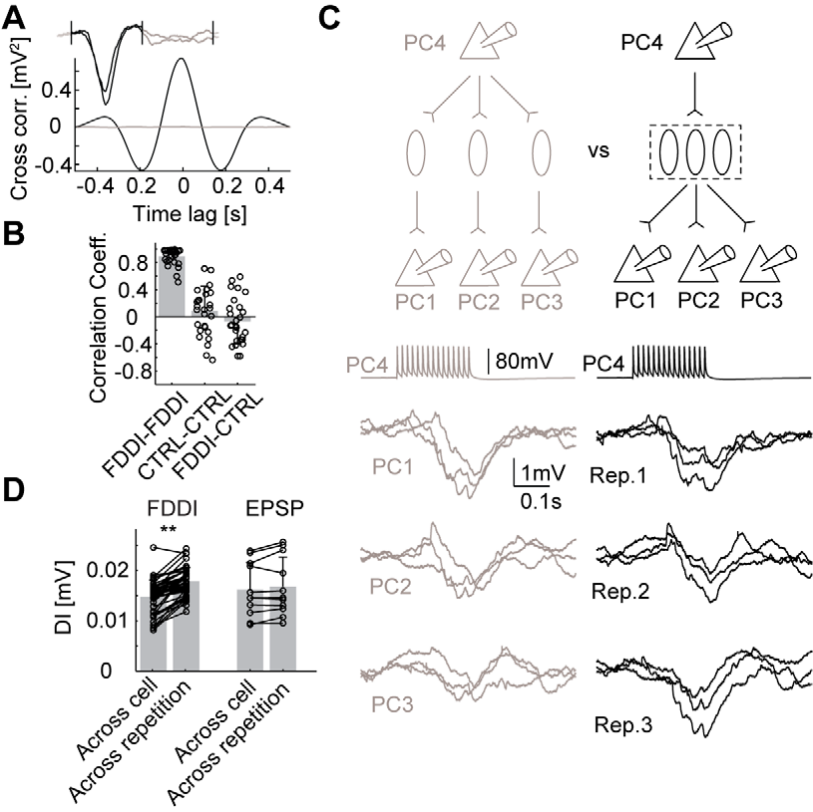
FDDI correlates subthreshold membrane potential in PCs. (**A**) Example of cross-correlation of averaged FDDI responses (black, time interval containing actual FDDI responses; gray, time interval 0.6 s after FDDI responses). Inset shows the averaged FDDI traces. (B) Correlation coefficient for simultaneously recorded FDDI responses in different PCs (FDDI-FDDI) and control conditions (CTRL-CTRL, correlation coefficient measured 0.6 s after FDDI response, see gray colored interval in the inset of (A); FDDI-CTRL, FDDI present in one PC but not in the other). (C) Sketch of experimental setup and stimulation protocol. Top, stimulation of PC 4 triggers FDDI in several postsynaptic PCs (1–3), which in principle could be mediated for each PC by a separate MC (PC-MC divergence, left, gray), or by one MC (or a pool acting as a functional unit) alone for all PCs (MC-PC divergence, right, black). Bottom, three responses following stimulation of PC 4, arranged according to the different cells (left, gray) and to the different repetitions (right, black). (D) Dissimilarity index (DI) between mean-subtracted traces for conditions across cell (different cells, same repetition) and across repetition (same cells, different repetition), showing for FDDI (left) higher trial-to-trial variability of identical PCs than PC-to-PC variability of the same trial. Divergent excitatory connections (EPSP, right) do not show this difference (see text for explanation). Error bars (B,D) denote s.d.

The high degree of interconnectivity between PCs and MCs results in subthreshold correlations between PCs (Figure 4A,B), showing a high correlation coefficient for simultaneous FDDI in different PCs (*n* = 28, μ_FDDI-FDDI_ = 0.892±0.125) and significantly lower ones for control conditions (*n* = 28, μ_CTRL-CTRL_ = 0.085±0.364, *n* = 26, μ_FDDI-CTRL_ = −0.070±0.331, *p*<0.00001, ANOVA with Scheffe correction). This correlation was calculated with average traces and is therefore based on mean responses. In order to estimate the similarity of FDDI in different PCs arising from stimulating a single PC, we performed a trial-to-trial analysis of divergent FDDI responses. In principle, divergent FDDI connectivity may be mediated by a high degree of divergence from PCs onto many different MCs and/or a high degree of divergence from MC to PCs (see Figure 4C for illustration). To quantify the amount of common FDDI input, we defined a “Dissimilarity Index” (DI), which is the root mean squared of mean subtracted traces (see Methods). DI was calculated pairwise between single trial traces, either between simultaneous traces of different cells, or, as a control, between traces of the same (or different, data not shown) cells but from different trials. If each postsynaptic PC received FDDI from a different set of interneurons (as illustrated in the left part of Figure 4C), the inhibitory response in the different postsynaptic PCs would not co-vary from trial to trial, resulting in a strong dissimilarity (high DI, as control). In contrast, if each postsynaptic PC received common input from the same set of interneurons (right part of Figure 4C), single-trial FDDI responses between different PCs should be more similar (low DI, smaller than control). If single-trial responses in PCs were identical, DI would be zero. In all tested cases except one, we found a lower DI of simultaneously acquired traces than that of non-simultaneously acquired traces, indicating a high degree of common MC input to neighboring PCs. The data of the illustrated example as well as 43 more cases suggest high MC to PC divergence (Figure 4D, *n* = 44, μ_ac_ = 0.0148±0.0033 mV, μ_ar_ = 0.0178±0.0028 mV, *p* = 2e-12, paired *t* test). Direct connections diverging from a PC to two or more postsynaptic PCs did not have a significantly different DI (*n* = 11, μ_ac_ = 0.0162±0.0054 mV, μ_ar_ = 0.0168±0.0058 mV, *p* = 0.1063, paired *t* test). These results show that divergent FDDI from a single PC onto multiple neighboring PCs is not because of a large set of MCs but can be accounted for by a highly divergent MC-PC connectivity. Combined with these findings on the contribution of a single MC on FDDI (Figure 3A–C), we conclude that the high prevalence of FDDI is supported by both PC-MC divergence as well as a high degree of MC-PC divergent connectivity. This MC-PC divergence causes the inhibitory inputs onto neighboring PC to be precisely timed and, together with the mean-based correlations (Figure 4B), enables FDDI to facilitate synchronization of PC activity.

**Figure 5:**
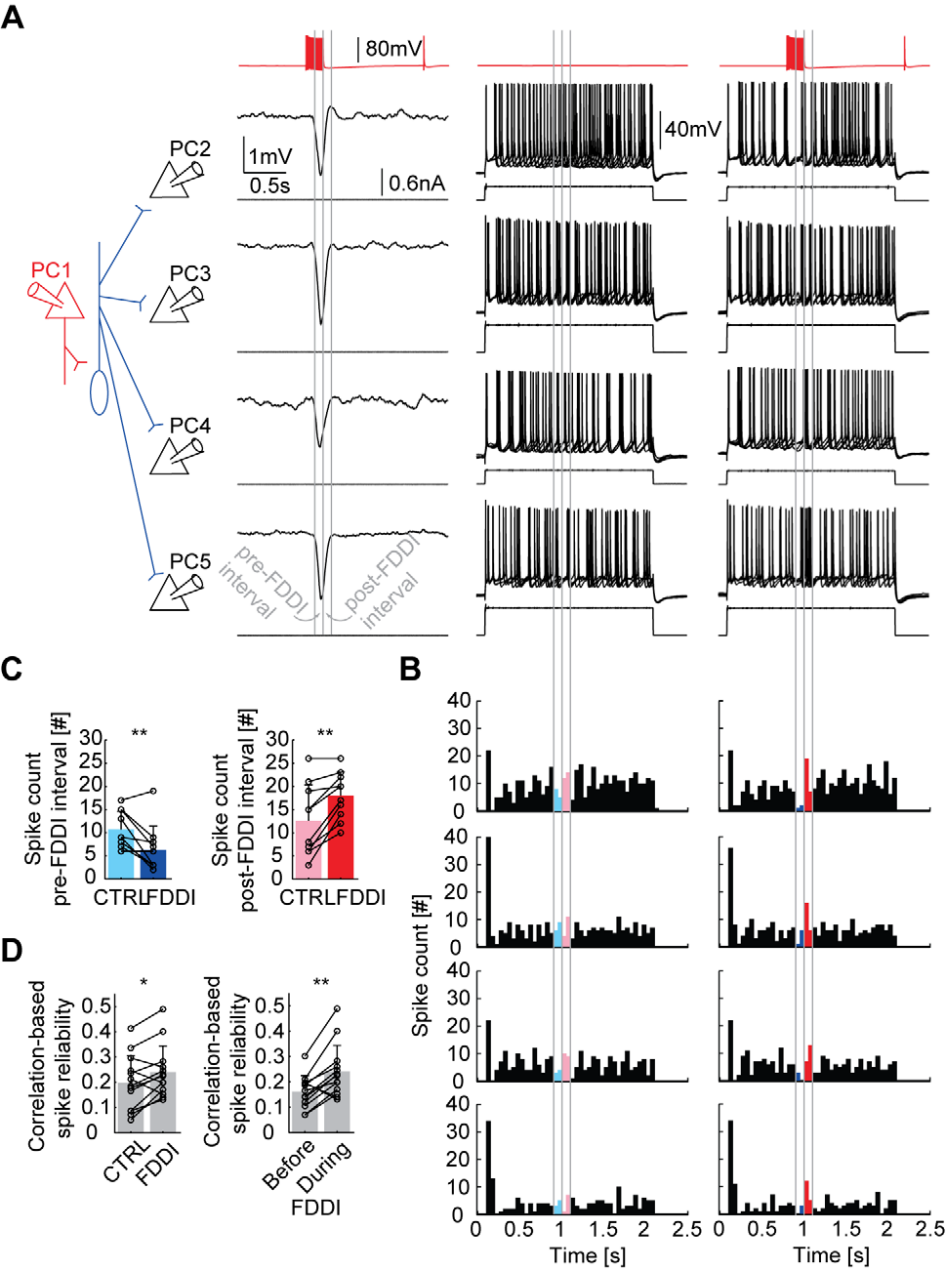
FDDI synchronizes spiking in PCs. (A) Sketch of the experimental setup and stimulation protocol. PC 1 (red) connects via one or multiple unpatched MCs to four postsynaptic PCs (black), eliciting FDDI in all of them (left column, traces show the average response of 22 repetitions). A suprathreshold step current of adjusted amplitude (200–600 pA) gave rise to low frequency spiking (2–8 Hz) with a certain jitter (middle column, for each cell five traces are shown). Both stimulations, FDDI and step current, synchronously applied led to a brief reduced (blue) and increased (red) spike rate during and at the offset of FDDI, respectively (right column). (B) Spike histograms for the control (left, only step current injection) and FDDI condition (right, both step current injection and FDDI). Bin size was 50 ms; note that 22 repetitions were applied. Same arrangement as in (A). (C) Spike count 100 ms before (left) and 100 ms after the peak of FDDI (right), during control and FDDI condition. (D) Correlation-based spike timing reliability with and without FDDI (left), or before and during FDDI (right). Time windows were 0.5 s long, from the onset of FDDI on or before the onset (control). Error bars (C,D) denote s.d.

Figure 5 shows this synchronization of multiple PCs in the suprathreshold regime. A single presynaptic PC (Figure 5A, red) was stimulated with high frequency (15 spikes at 70 Hz) and elicited FDDI in multiple postsynaptic PCs (black, left column). Postsynaptic PCs were stimulated with a suprathreshold step current (resulting in low frequency spiking of 2–8 Hz) in the presence of FDDI input (right column), and as a control, without FDDI input (middle column). Without FDDI input, firing of PCs already displayed some variability from trial to trial, probably due to spontaneous membrane potential fluctuations and drifts over the long duration of the stimulus paradigm. As can be seen in the peristimulus time histogram (Figure 5B), however, the probability of spiking is reduced during the beginning of FDDI (blue color), followed by a period of “rebound spiking” at the end and briefly after FDDI (red color). We quantified this effect by counting spikes during this first (left part of Figure 5C, *n* = 11, μ_CTRL_ = 10.7±3.92, μ_FDDI_ = 6.3±5.1, *p* = 0.0048, paired *t* test) and second 100 ms time window (right part of Figure 5C, μ_CTRL_ = 12.6±7.7, μ_FDDI_ = 18±5.1, *p* = 0.0015, paired *t* test) of 22 repetitions in control and FDDI condition. This effect is also quantified by a correlation-based spike timing reliability measure (Figure 5D; standard deviation of the Gaussian used for convolution with the spike trains was 10 ms; for details on the method, see [23]). Spike timing reliability between single repetitions of pairs of postsynaptic PCs increases during FDDI (left part of Figure 5D, *n* = 18, μ_CTRL_ = 0.197±0.109, μ_FDDI_ = 0.241±0.1034, *p* = 0.018, paired *t* test), which also holds true if a time window before FDDI onset is chosen as a control (right part of Figure 5D, see methods, μ_BEFORE_ = 0.162±0.064, μ_DURING_ = μ_FDDI_, *p* = 0.003, paired *t* test). Thus, FDDI can lead to synchronous pauses followed by subsequent synchronous spiking in neighboring PCs.

### Diversity of FDDI Summation and Cooperativity

**Figure 6:**
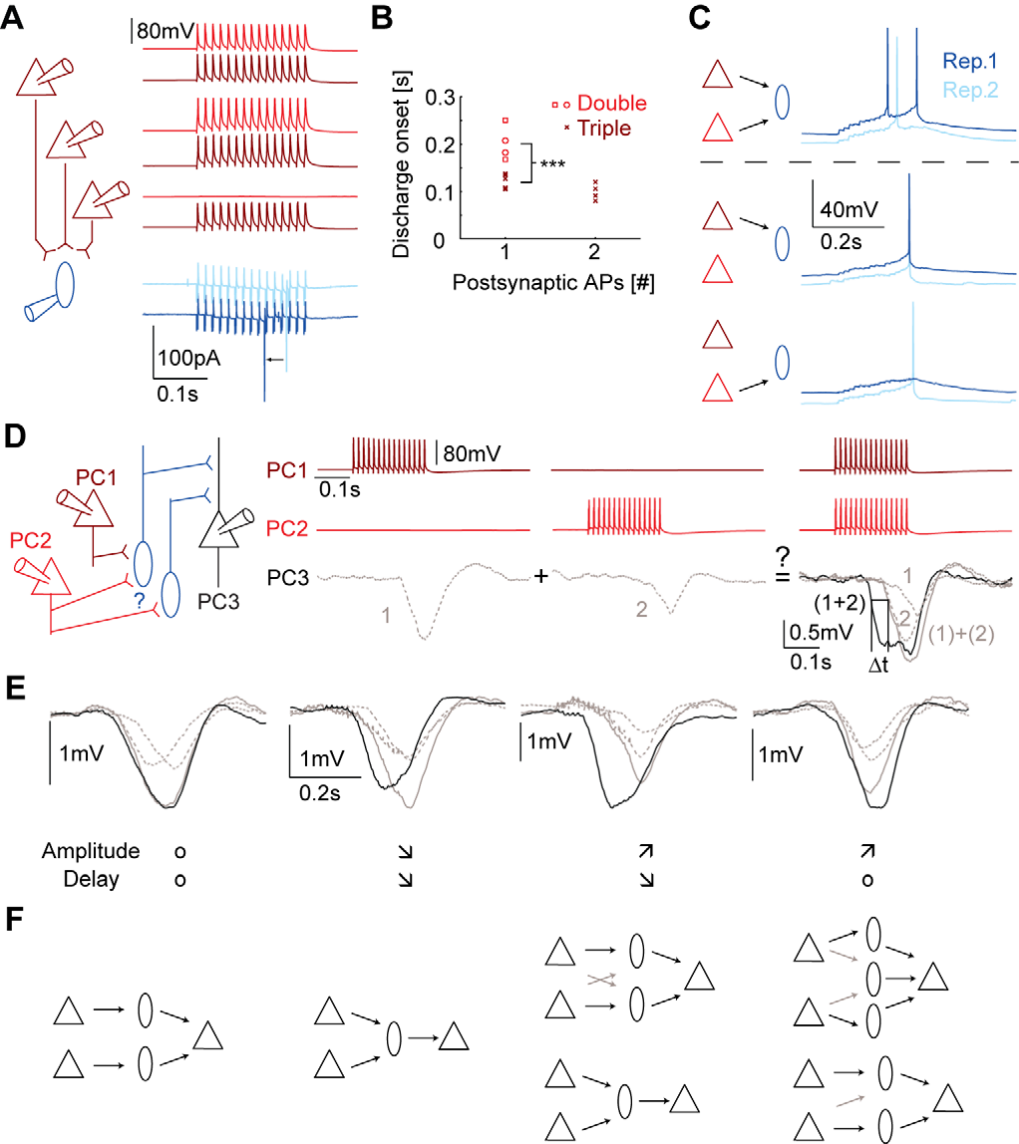
Summation properties indicate network configuration. (A,B) PC-MC convergence leads to a reduced latency of MC spiking and increases number of MC spikes. (A) Two out of three (red traces) or three out of three PCs (dark red), all presynaptic to an MC recorded in the cell-attached mode, were stimulated with a high frequency (15 APs at 70 Hz). Stimulation of three PCs led to a reduced delay in the spiking response of the MC. (B) Discharge onset during stimulation of two (red, double) or three PCs (dark red, triple). Stimulation of three PCs occasionally led to two postsynaptic APs in the MC. (C) Stimulation of two PCs converging onto a postsynaptic MC (recorded in the whole-cell mode) can lead to more and earlier APs in the MC (blue traces) or to an earlier single AP only (light blue traces) as compared to single PC stimulation. (D–F) Summation properties of FDDI partially explain underlying connectivity pattern. (D) Sketch of experimental setup and stimulation protocol. High frequency stimulation (15 APs at 70 Hz) in PC 1 or PC 2 with corresponding FDDI in PC 3 (left or middle column), and simultaneous stimulation of PC 1 and PC 2 with FDDI response in PC 3 that has an earlier onset (right column). (E) Different scenarios of summation showing either no difference, reduced delay with reduced amplitude, or increased peak amplitude with and without reduced delay (same color code as in (D)). (F) Underlying connectivity patterns that can explain the summation properties. Black arrows denote suprathreshold connections, gray arrows subthreshold connections. Note that these connectivity schemes show the simplest scenarios, with the minimal number of mediating MCs needed (see Results).

**Figure 7 pbio-1000473-g007:**
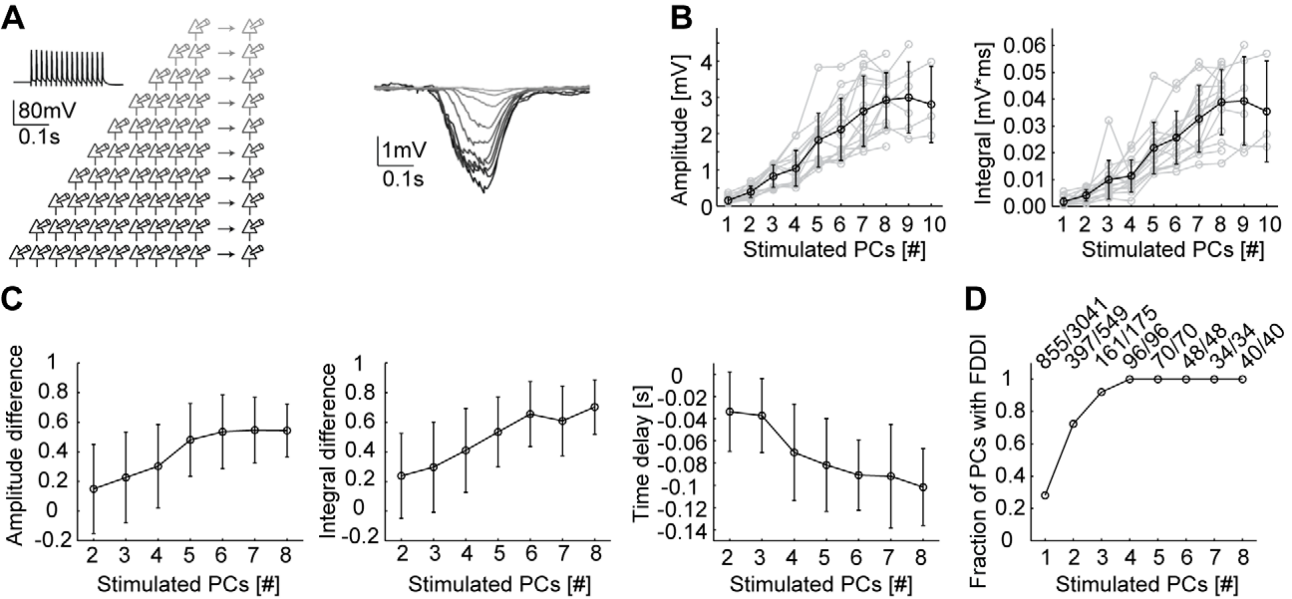
FDDI saturates at resting conditions with few stimulated PCs. (A) Sketch of experimental setup and example traces. Up to 10 cells were stimulated simultaneously (left). FDDI in response to 1–10 PCs stimulated (15 APs at 70 Hz, right). (B) FDDI amplitude and integral as a function of the number of PCs simultaneously stimulated. (C) Saturation of FDDI as a function of the number of stimulated PCs for amplitude and integral difference and time delay. (D) Fraction of PCs displaying FDDI as a function of number of PCs stimulated. Error bars (B, C) denote s.d.

Next, we investigated the spatial and temporal integration properties of FDDI in a single PC when multiple presynaptic PCs are stimulated at the same time. Figure 6A shows that an increased number of stimulated PCs leads to a reduced delay of MC firing. The MC was recorded in cell-attached mode so that the intracellular medium remained undisturbed. Not only does the discharge onset take place earlier by tens of milliseconds (μ_3pre_ = 0.114±0.021 s, μ_2pre_ = 0.202±0.036 s, *p* = 0.000021, two-sample *t* test), but also the number of APs fired by the MC increases (Figure 6B). Figure 6C shows the same type of experiment with the MC recorded in whole-cell mode. Stimulation of two PCs simultaneously can lead to earlier and more numerous spikes (Rep. 1, blue traces), a PC-MC convergence configuration, which would lead to earlier and larger FDDI in a PC postsynaptic to the MC (supralinear summation). On the other hand, simultaneous stimulation may also lead to earlier MC spiking only (Rep. 2, light blue traces), which should lead to reduced FDDI amplitude in postsynaptic PCs (sublinear summation). In view of the latency shortening and increased discharge in MCs, we analyzed both amplitudes and onset latencies of FDDI mediated by several presynaptic PCs onto a single postsynaptic one (Figure 6D–F, Figure 7).

Similarly to the previous summation experiments we compared FDDI in response to synchronous stimulation of two PCs (Figure 6D, black traces in the right column) to the off-line calculated sum of the separate stimulations (left and middle column, gray dashed traces, and gray traces in right column). As expected, a variety of different responses were found (Figure 6E), ranging from linear summation (left), reduced amplitude with reduced onset delay (left middle), increased amplitude with reduced onset delay (right middle), and increased amplitude with same delay (right). Possible underlying connectivity schemes are depicted in Figure 6F. In cases where the onset delay was shortened, it is very likely that the FDDI is mediated by MCs receiving convergent common excitation from both PCs. The common input decreases the discharge onset of the MC(s) and results in earlier onset of inhibition (see Figure 6A–C). Networks that did not exhibit a latency decrease following co-stimulation may also involve MCs receiving common input but not exclusively (Figure 6F, right). The origin of amplitude summation is more complicated, since both supra- and sublinear summation can be explained by convergent PC-MC inputs: if an intermediate MC can be reliably activated only by convergent input, this will result in an average supralinear increase in amplitude. However, if an MC discharges reliably following inputs from both PCs individually, such that co-activation does not significantly increase the number of APs, the result is sublinear amplitude summation. Our results show that on average the latency was shortened by 33.7±35.8 ms (*n* = 103, *p*<0.0001, two-tailed *t* test), and the amplitude increase was supralinear (μ_sync_ = 1.232±0.723 ms, μ_summed_ = 1.111±0.877 mV, *n* = 103, *p* = 0.00096, paired *t* test). These results indicate that co-stimulation of presynaptic PC pairs increases FDDI in a supralinear manner due to the high degree of PC-MC convergence.

### Few PCs Saturate FDDI

How does FDDI summate when more than two neighboring PCs are active? We stimulated an increasingly larger number of PCs and recorded FDDI in another PC (Figure 7A, gray shades of the traces according to the number of stimulated cells). FDDI monotonically increased in amplitude and voltage integral, which saturated when eight to nine PCs were simultaneously stimulated (Figure 7B). In order to compare the pooled data of many recorded clusters, we used a nonlinearity index for amplitude and integral summation [15]. FDDI summated on average supralinearly for the amplitude as well as for the integral following stimulation of two presynaptic PCs (Figure S1, *n* = 103, p_amp_ = 1.977e-6, p_int_ = 2.710e-13, one-sample *t* test). Stimulation of three or more (Figure 7C and S1) presynaptic PCs increased the supralinearity of integral and amplitude of FDDI, and also decreased the onset delay. Saturation levels of amplitude and integral difference were reached at around 60% and 70% when six to seven PCs were stimulated simultaneously (Figure 7C). A remarkable feature of FDDI was its abundance in the layer 5 network. Upon stimulation of four PCs simultaneously, *all* recorded neighboring PCs were inhibited (Figure 7D).

### Brief Bursts Can Trigger FDDI

**Figure 8:**
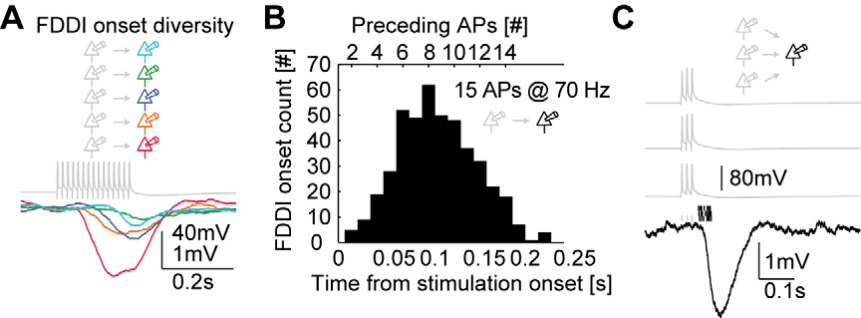
Brief bursts can trigger FDDI. (A) PCs (of clusters in different experiments) were stimulated with 70 Hz spike trains, resulting in FDDI in postsynaptic PCs with various onset latencies. Traces are mean responses of 20–30 repetitions. In the given examples, FDDI has been triggered after 5, 6, 8, 11, and 12 APs, respectively. (B) Histogram of FDDI onset as a function of time since stimulation onset and number of preceding presynaptic APs. FDDI in a postsynaptic PC was triggered by stimulating a single presynaptic PC with 15 APs at 70 Hz. (C) FDDI in a postsynaptic PC (black trace) triggered by three presynaptic PCs (gray traces) stimulated with three APs at 70 Hz. Trace is a single iteration. Gray vertical bars above the response trace indicate spike times, and black vertical bars indicate FDDI onsets of all recorded cases (vertically separated in three rows for better visibility).

Our typical stimulation protocol used to elicit and reliably identify FDDI contained multiple APs (15) and high frequencies (70 Hz), a condition that is presumably unlikely to be experienced by PCs in the intact brain. However, the onset of FDDI after this long-train stimulation is variable between cells (Figure 8A), and in several cases, less APs would have been sufficient to trigger FDDI by a single PC, since the hyperpolarization can start off briefly after stimulus onset (Figure 8B, *n* = 439, mean = 0.110 s, eight presynaptic APs). We also know that synchronous activation of multiple PCs can significantly decrease the FDDI onset (Figures 6, 7 and S1). In order to examine whether FDDI can be triggered with few APs only, we stimulated three presynaptic PCs with only three APs at 70 Hz, mimicking the spiking output evoked by dendritic calcium spikes [24]. As can be seen in Figure 8C, even this condition is sufficient to elicit FDDI reliably, with a probability of occurrence of 0.23 (23 out of 99 tested different quadruplet combinations of cells), a mean onset delay of 0.068±0.012 s, and amplitudes of up to several millivolts (μ = 1.22±0.77 mV; range 0.25–3.7 mV). This illustrates that brief synchronous bursts (∼three APs at 70 Hz) of only three PCs are able to trigger FDDI in neighboring PCs, a condition that is likely to be relevant in the in vivo situation.

## Discussion

This study reveals the key properties of one of the physiologically and anatomically most distinguished disynaptic inhibitory pathways in the neocortex, FDDI: the number of PCs required, divergent and convergent properties to and from MCs, spatio-temporal principles that govern the integration of the inhibition applied through this pathway, the dependency of this form of inhibition on I_h_ currents, and its potential influence on the functioning of the network of thick tufted PCs in the somatosensory neocortex of juvenile rats.

### Summation, Saturation, and Limits of FDDI Recruitment

Previously, the summation properties for convergent FDDI have been investigated for two [15] or three [14] stimulated presynaptic PCs, and the activity-dependent recruitment of MCs was extrapolated for the case of multiple active PCs [15]. Two of our most important findings are that (a) every neighboring (<150 µm intersomatic distance) thick tufted layer 5 PC is affected by FDDI when four or more PCs burst simultaneously and that (b) the FDDI amplitude saturates at the somatic recording site at resting condition when eight to nine PCs are stimulated simultaneously. We find the low number of PCs necessary to trigger FDDI in all neighboring PCs especially remarkable—it shows that in the high frequency, high correlation range the major signaling between PCs is (after an initial brief excitatory response) inhibitory. The observed FDDI saturation may be caused by several reasons: limited recruitment of MCs (due to limited connectivity or limited number of MCs), reduction in the driving-force of the inhibitory signal in the apical dendrite when it reaches the GABA_A_ reversal potential, saturating firing rates in MCs, and frequency-dependent synaptic depression of the MC-PC connection. It is likely that all these factors contribute to this early saturation.

Summation properties as shown in Figures 3, 4, and 6 indicate that only a few MCs are actually recruited by a cluster of PCs. We cannot, however, state that MC *recruitment* is saturated by stimulation of eight to nine PCs since multiple MCs could mutually shunt their inhibitory signals in a postsynaptic PC and therefore mask the contribution of additional MCs to FDDI. Further, it should be considered that the saturation might not hold in different cortical activity states. For example, if reduced driving force was a major reason for saturation at rest, FDDI might saturate at a later stage at more active cortical states. A high level of excitatory synaptic input to the apical dendrite would require larger activation of the FDDI pathway in order to reach saturation. Kapfer and colleagues [15] extrapolated PC-PC and PC-MC connectivity data to predict a saturation curve for MC recruitment (see Figure 6 therein). Although the studies are not directly comparable and were performed in different cortical layers, our data suggest a smaller dynamic range for recruitment of inhibition and earlier saturation than previously reported. What is not exactly known and not addressed in the current study is the degree of synchrony that brief bursts of neighboring need to have in order to trigger FDDI. We only tested simultaneous stimulations of PCs; a jitter in the PC firing is likely to alter the efficiency of FDDI recruitment and amplitude.

### Subthreshold Correlations between PCs

Neighboring neocortical cells can show highly correlated activity patterns both in vitro [25] and in vivo [26],[27]. Recently, it has been shown that the synchrony of subthreshold membrane potential fluctuations depends on the behavioral state of the animal [28]. FDDI acts as a synchronizer of subthreshold membrane potential between PCs in two ways. Multiple PCs, targeted by the same MC, receive FDDI simultaneously, resulting in a high correlation coefficient (Figure 5B). Moreover, due to the reliability of the MC-PC synapse, and its high divergence, the inter-trial variability is mainly due to the summation of the facilitating PC-MC synaptic response. A previous study has also shown that the synaptic dynamics from interneurons are virtually identical across postsynaptic neurons of the same class, which may also underlie the high subthreshold correlations mediated by MCs [29]. Simultaneous responses in different postsynaptic PCs are therefore more similar to each other than the responses of the same PC for different iterations. The high correlation in FDDI across PCs suggests that inhibitory inputs from MCs to PCs may contribute to subthreshold correlations observed between neighboring PCs under in vivo conditions [26],[27]. Photostimulation studies have suggested that interneurons with adapting firing pattern (like MCs) are less specific or selective concerning the targeting of their synaptic input and output [30], a finding which is in agreement with the high degree of FDDI divergence we report (Figure 5A).

### Inhibition in the Pyramidal Network

Two dynamically different disynaptic inhibitory pathways have been identified in the neocortex [14] and their equivalents in the hippocampus [31]. The pathways differ in their dynamical as well as morphological properties, with the delayed, frequency-dependent pathway activated by MCs (belonging to the low threshold spiking (LTS) class of interneurons), triggered by facilitating connections from PCs and target PC dendrites. The other inhibitory pathway conversely is “immediate” and time-locked to PC single APs. It is mediated by depressing connections onto fast-spiking cells, typically PV-expressing basket cells, which in turn target PC perisomatic regions. These interneurons also mediate strong feed-forward inhibition activated by the thalamocortical pathway that has received attention in recent studies, showing that FS interneurons respond to thalamic input by discharge that precedes that of their excitatory neighbors [32]–[34]. LTS cells, on the other hand, receive only weak thalamic input [34],[35] (but see [36]), suggesting that their activation is primarily intracortical, optimally driven by high-frequency burst discharge of PCs.

One implication of the dendritic locus of MC-PC connections, reaching up to the distal dendritic tuft [14], suggests that FDDI has a role in regulating dendritic excitation, including intrinsic excitability in the form of calcium [24],[37] and NMDA spikes [38]. Indeed, in a recent study, Murayama and colleagues [39] demonstrated direct blocking of dendritic calcium spikes by FDDI in older animals (24–40 d old), showing that FDDI is preserved in development and can regulate dendritic excitability in layer 5 PCs. The authors also showed that GABAergic inhibition to PC dendrites originated from layer 5 interneurons and was crucial for enabling a wide dynamic range of calcium responses in vivo, correlated to the intensity of sensory stimulus. Therefore, FDDI might be a precisely matching antagonist of active excitatory conductances like calcium spikes, both being triggered by high frequency bursts. Our study was performed in younger animals, suggesting that development of FDDI onto PC dendrites precedes the maturation of their excitability, which occurs after the third postnatal week [40].

### Modulation of FDDI

I_h_ is a prominent current with increasing channel density along the dendrites of layer 5 PCs [41],[42]. It renders the apical (and presumably also the basal) dendrites disconnected from the soma [43] by counteracting any polarization deviating from the resting potential. The decay times of de- and hyperpolarizing inputs are substantially shortened, allowing for a higher temporal precision in the processing of information. Due to its increasing density along the dendrites it also renders EPSP shape and time course site independent [44]. Here we showed that I_h_ can change the gain between excitation and inhibition for train stimulations, thus increasing the dynamic frequency range. A modulation of this channel conductance might be an approach to profoundly alter this inhibitory pathway [42],[43]. Studies showed I_h_ presence in MCs as well, but there seem to be exceptions to this finding, with not all MCs expressing the I_h_ mediated sag in response to hyperpolarizing step currents [18]. We also did not find a prominent sag in MCs that participated in FDDI (*n* = 3, see also Figure 3C). The relative contribution of the various MC populations to FDDI remains to be elucidated [45].

MCs can be modulated by various means. Acetylcholine receptor agonists lead to increased firing in MCs [46], which might influence the plasticity rules at the apical dendrite of PCs [47]. LTS cells, have been shown to synchronize and oscillate in response to a G-protein coupled glutamate receptor antagonist [48]. This synchronization, mediated by electrical synapses, should enhance and broaden the effect of FDDI in the PC population. Compared to other cell types, MCs seem to be particularly susceptible to changes in the general cortical activity state [49]. Spiking activity (in certain frequency ranges) in MCs can trigger intracellular endocannabinoid signaling that eventually leads to hyperpolarization, and thus reduced excitability [50],[51]. It remains to be elucidated which modulations play strong roles under physiological conditions and to what extent FDDI properties reported in the present study are altered.

### FDDI in Other Cell Types

Several aspects of FDDI still remain to be elucidated. So far, layer 5 thick tufted PCs and layer 3 PCs have been shown to display FDDI [14],[15]. Cortical-callosal layer 5 PCs with a slender apical dendrite lacking tuft dendrites do not seem to feature this type of inhibition [52]. Also, PCs in layer 6 do not show any measurable FDDI (Berger and Markram, unpublished data). It is, however, not clear whether these potential pathways require a larger number of active neurons to become observable. It remains to be shown whether other PC classes are inhibited in a similar manner and whether this inhibition is mediated via the same MCs. Apart from the FDDI mediated within the same layer, it is possible that presynaptic activity in one layer will inhibit PCs in a different layer. Kapfer and colleagues showed that MCs in layer 5 mediated FDDI between layer 3 PCs [15], which is in agreement with the neurons' axonal terminal distribution [18],[45]. It is not known, however, if these MCs are the ones that also mediate FDDI onto layer 5 PCs and whether they are also recruited by layer 5 PCs. It also remains to be elucidated whether supragranular MCs also participate in FDDI between layer 5 PCs. Layer 5 PCs do innervate layer 2/3, and these MCs target preferentially supragranular layers and possibly also the apical trunk or tuft of layer 5 PCs. Subpopulations of SOM expressing interneurons are now GFP labeled in various mouse strains [45], facilitating future investigations of FDDI in different layers. A recent study described differences in monosynaptic excitatory connectivity between different types of layer 5 PCs [53], according to their long-rage projections. It would be of great importance to determine the properties of disynaptic inhibition between these populations as well.

## Methods

### Slice Preparation and Cell Identification

Fourteen- to 18-d-old Wistar rats (mean age 15.0 d, range 14–18 d) were quickly decapitated according to the Swiss national and institutional guidelines. The brain was carefully removed and placed in iced artificial cerebrospinal fluid (ACSF). Three hundred µm thick parasagittal slices of the primary somatosensory cortex (hindlimb area) were cut on a HR2 vibratome (Sigmann Elektronik, Heidelberg, Germany). Slices were incubated at 37°C for 30–60 min and then left at room temperature until recording. Cells were visualized by infrared differential interference contrast videomicroscopy utilizing either a C2400-03 camera (Hamamatsu, Hamamatsu City, Japan) mounted on an upright Axioscope FS microscope (Zeiss, Oberkochen, Germany) or a VX55 camera (Till Photonics, Gräfeling, Germany) mounted on an upright BX51WI microscope (Olympus, Tokyo, Japan). Thick tufted layer 5 PCs were selected according to their large soma size (15–25 µm) and their apparent large trunk of the apical dendrite. Care was taken to use only “parallel” slices, i.e. slices that had a cutting plane parallel to the course of the apical dendrites and the primary axonal trunk. This ensured sufficient preservation of both the PCs' and MCs' axonal and dendritic arborizations. Some experiments included recording of MCs. They were targeted by their soma, which is oval and bitufted, and often oriented sideways.

### Chemicals and Solutions

Slices were continuously superfused with ACSF containing (in mM) 125 NaCl, 25 NaHCO3, 2.5 KCl, 1.25 NaH_2_PO_4_, 2 CaCl_2_, 1 MgCl_2_, and 25 D-glucose, bubbled with 95% O_2_–5% CO_2_. The intracellular pipette solution (ICS) contained (in mM) 110 K-gluconate, 10 KCl, 4 ATP-Mg, 10 phosphocreatine, 0.3 GTP, 10 N-2-hydroxyethylpiperazine-N9-2-ethanesulfonic acid (HEPES), and 13 biocytin, adjusted to a pH 7.3–7.4 with 5 M KOH. Osmolarity was adjusted to 290–300 mosm with D-mannitol (25–35 mM). The membrane potential values given were not corrected for the liquid junction potential, which was approximately −14 mV. 4-(*N*-ethyl-*N*-phenylamino)-1,2-dimethyl-6-(methylamino) pyridinium chloride (zd7288) was bought from Biotrend (Zurich, Switzerland), and all other drugs and chemicals were from Sigma-Aldrich (St. Louis, MO) or Merck (Darmstadt, Germany).

### Electrophysiological Recordings

Multiple somatic whole cell recordings (2–12 cells simultaneously) were performed with Axopatch 200B or Multiclamp 700B amplifiers (Molecular Devices, Union City, CA) in the current clamp mode. We selected PCs that were located close to each other, preferentially in clusters of near to adjacent cells. When 12 cells were recorded at the same time, the pairwise intersomatic distance increased due to limited accessibility with multiple patch electrodes in the tissue but did not exceed 150 µm. In some experiments, MCs were first recorded in voltage clamp in the cell-attached configuration, leaving the intracellular medium unperturbed, and then in whole-cell mode, thus perfused with the ICS and Biocytin contained in the pipette, allowing a subsequent staining and cell type identification. In experiments including dendritic recordings, dendrites were patched before the somata. Alexafluor594 (Invitrogen, Eugene, OR) was sometimes included in the dendritic patch electrode, revealing the corresponding soma unambiguously. The temperature was 34°C±1°C during recording. Data acquisition was performed via an ITC-18 or ITC-1600 board (Instrutech Co, Port Washington, NY), connected to a PC or Macintosh running a custom written routine under IgorPro (Wavemetrics, Portland, OR). Sampling rates were 5–10 kHz, and the voltage signal was filtered with a 2 kHz Bessel filter. Patch pipettes were pulled with a Flamming/Brown micropipette puller P-97 (Sutter Instruments Co., Novato, CA) and had an initial resistance of 3–8 MΩ (10–15 MΩ for dendritic patches).

3D morphological reconstruction of biocytin-labeled cells was done under an Olympus BX 51 W microscope fitted with a water-immersion 60× (numerical aperture (NA) 0.9) or an oil-immersion 100× (NA 1.35) objective using Neurolucida software (MicroBrightField, Magdeburg, Germany).

### Stimulation Protocols and Data Analysis

Monosynaptic, direct connections were usually identified by stimulation of a presynaptic cell with a 20 Hz train of eight strong and brief current pulses (1–3.5 nA, 2–4 ms), followed by a so-called recovery test response (RTR) 0.5 s after the end of the train, all precisely and reliably eliciting APs. Disynaptic connections were characterized by the same protocol but at a higher frequency (usually 70 Hz) and with longer trains (usually 15 APs). Postsynaptic PCs were slightly depolarized from a potential of ∼−62 mV to −57 to −60 mV to increase the driving force for inhibitory connections. This was usually not necessary to detect FDDI but gave larger amplitudes occasionally. Due to the dendritic location and the resulting space clamp effect in layer 5 PCs, especially MC-PC synapses have a very hyperpolarized apparent somatic reversal potential that deviates strongly from the calculated one [14]. We did not find any depolarizing FDDI responses, possibly because we used rats older than 13 d [54]. Connectivity ratios were calculated as the ratio between observed versus tested connections between a pair of cells. A pair of cells could therefore maximally have two connections (both directions), a triplet could have six connections, and a cluster of *n* neurons could potentially have *n* * (*n*−1) connections. “Autaptic” connections—that is, FDDI elicited and received by the same PC—were not taken into consideration.

The balance between de- and hyperpolarization due to FDDI and direct EPSPs as a function of stimulation frequency (Figure 1E) was calculated as the net polarization deviating from baseline in the time window starting from stimulation onset and ending just before the RTR, i.e. 0.5 s after the stimulation train ended. Bath application of zd7288 resulted in a strong hyperpolarization of PCs (∼10–12 mV; [14]), which was counteracted by a positive holding current to reestablish resting membrane potential of around −60 mV. The waiting time between stimulations was 10–20 s. Especially for FDDI summation experiments (Figures 6–8, S1) long waiting times were crucial as FDDI amplitudes would decrease otherwise (much more dramatic than, e.g., EPSP amplitudes). For these figures, we only included “pure” FDDI responses (without monosynaptic EPSP contamination) in the analysis. Stimulations were given in an alternating manner (ABAB… instead of AABB…). For summation experiments as shown in Figure 7 and S1, linearity of amplitude (and likewise integral) was calculated as a normalized difference according to L = (A_input(1,2,…,*n*)_−(A_input1_+A_input2_+…+A_inputn_))/A_input(1,2,…,*n*)_, where A_input(1,2,…,*n*)_ is the amplitude of the simultaneous stimulation and A_input1_+A_input2_+…+A_inputn_ is the offline calculated sum of the separately stimulated presynaptic cells. For Figures 7C,D and S1, data were included if the FDDI evoked by synchronous stimulation exceeded 0.5 mV, as the signal-to-noise ratio was too high for the difference measures otherwise. All statistical analysis (paired and unpaired student's *t* test, ANOVA) was done with MATLAB (The Mathworks, Natick, MA, USA). The DI was defined as
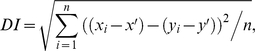
where x_i_ and y_i_ are single repetitions of baseline-subtracted (mean of the first 100 ms before stimulation was taken as a reference) traces of different or identical cells and different or identical repetitions, and x′ and y′ are the baseline-subtracted mean responses. DI is the point-wise squared difference between mean- and baseline-subtracted traces, calculated for every possible pair of traces, i.e. “across cells, same repetition,” “same cell, across repetitions,” and “across cells, across repetitions.” It quantifies the deviation from the average response and shows whether noise coming along the FDDI signal co-varies between two cells or not. Given one stimulated presynaptic PC, two postsynaptic PCs receiving FDDI, and *n* repetitions of stimulation, one obtains *n* “across cells, same repetition” conditions, *n* * (*n*+1)/2 “same cell, across repetitions” conditions, and *n* * (*n*−1) “across cells, across repetitions” conditions. For the latter two conditions the DI measure was nearly identical, therefore the “across cells, across repetitions” condition is not displayed in Figure 4E. DI was taken for the interval from 0 to 0.5 s after stimulation onset. Note that DI is intended to compare traces that have been stimulated in the same way. It is therefore not meaningful to compare DI values of FDDI (stimulated with 15 APs at 70 Hz, disynaptic) with EPSPs (8 APs at 20 Hz, monosynaptic). Cross-correlation and Pearson's correlation coefficient of two mean FDDI responses (Figure 4A and 4B) were calculated with Igor Pro. The effect of FDDI on spiking postsynaptic PCs (Figure 5) was quantified by counting spikes at specific 100 ms time windows of the peristimulus time histogram, namely during the second half of (first window) and immediately after (second window) presynaptic train stimulation. Spiking responses of postsynaptic PCs without coincident FDDI input served as control condition. Peristimulus time histograms contained spike counts of around 22 repetitions. Correlation-based spike timing precision was calculated according to [23] and on 0.5 s long time windows.

## Supporting Information

Figure S1 **Summation properties of FDDI elicited by two to eight presynaptic PCs.** Histograms show amplitude and integral difference as well as the time delay between the response of a PC to two to eight synchronously stimulated PCs and their offline summated, separately stimulated responses. A positive amplitude (integral) difference means that synchronous stimulation of the two PCs gave a larger FDDI amplitude than the offline summed response of the individually evoked FDDIs. A more negative time delay shows an earlier response of the synchronously evoked FDDI as compared to the summed response of the individually evoked FDDIs.
(1.02 MB TIF)

## Abbreviations

DI: Dissimilarity Index
EPSP: excitatory postsynaptic potential
FDDI: frequency-dependent disynaptic inhibition
IPSP: inhibitory postsynaptic potential
LTS: low threshold spiking
MC: Martinotti cell
PC: pyramidal cell
