# Supplementary figures and images for: Brief Bursts Self-Inhibit and Correlate the Pyramidal Network
Source: PLoS Biol. 2010 Sep 7;8(9):e1000473. doi: 10.1371/journal.pbio.1000473 (PMC2935452; doi:10.1371/journal.pbio.1000473)

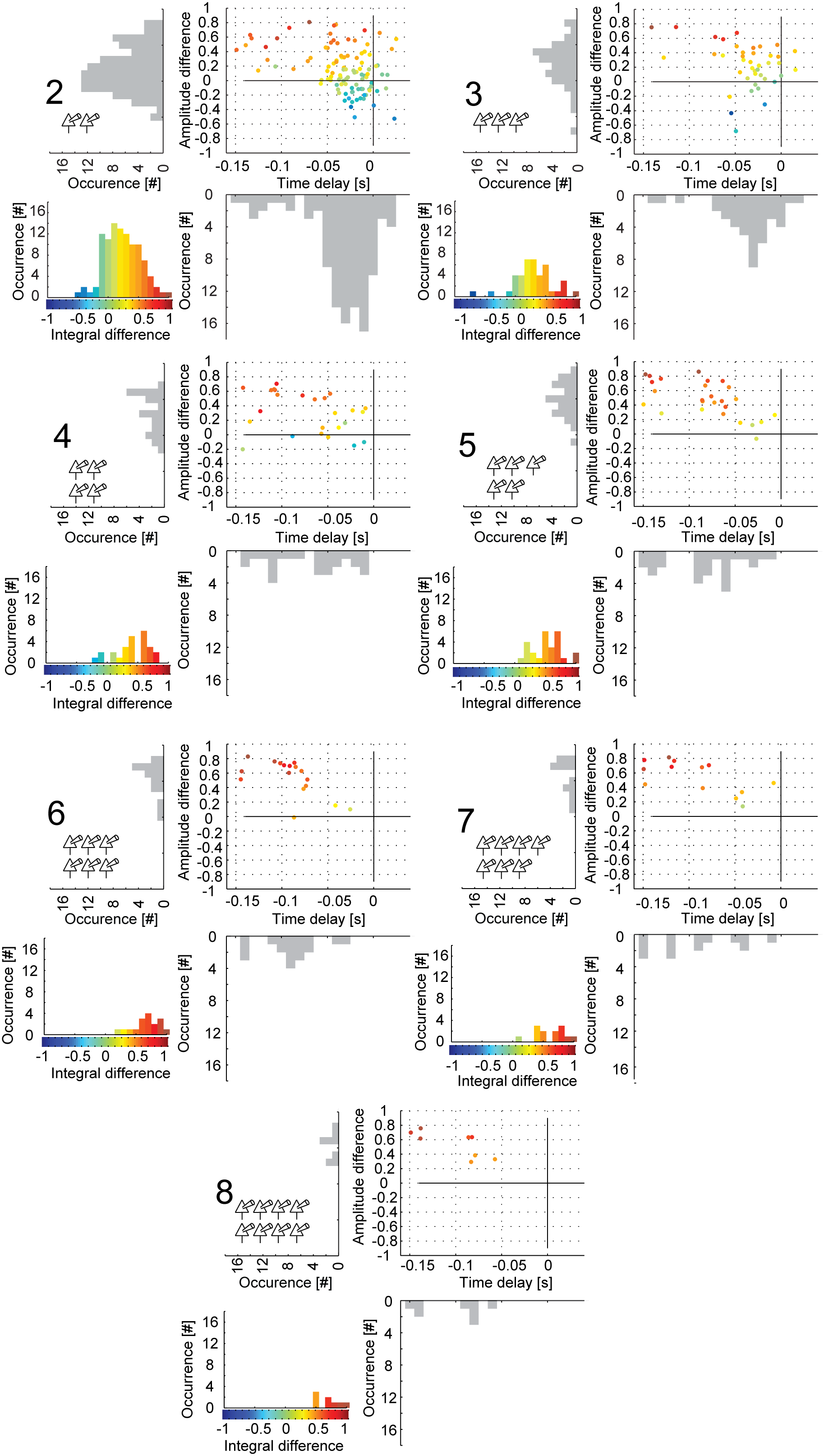

Supplement: Figure S1 — Summation properties of FDDI elicited by two to eight presynaptic PCs. Histograms show amplitude and integral difference as well as the time delay between the response of a PC to two to eight synchronously stimulated PCs and their offline summated, separately stimulated responses. A positive amplitude (integral) difference means that synchronous stimulation of the two PCs gave a larger FDDI amplitude than the offline summed response of the individually evoked FDDIs. A more negative time delay shows an earlier response of the synchronously evoked FDDI as compared to the summed response of the individually evoked FDDIs. (1.02 MB TIF) [file pbio.1000473.s001.tif]
